# Supplementary material for: Complexome profiling of the Chlamydomonas psb28 mutant reveals TEF5 as an early PSII assembly factor
Source: Plant Cell. 2025 Mar 17;37(6):koaf055. doi: 10.1093/plcell/koaf055 (PMC12164590; doi:10.1093/plcell/koaf055)
Supplement: koaf055_Supplementary_Data [file koaf055_supplementary_data.zip › psb28_interactive.zip!/plots/intensity/qualityPlots/peptides/PSRP1_Cre05.g237450.t1.2.html]

### Cre05.g237450.t1.2

Trivial name: PSRP1  
SeqCoverage [median]: 41.80  
Mass: 31.71 kDa  
  
 

| Sequence | MissedCleavage | Mass [Da] | wtIntensity | muIntensity | Score |
| --- | --- | --- | --- | --- | --- |
| AGPKEDVDEEDFQEYLK | 1 | 2010.91 | 0.00 | 6083.52 | 104.44 |
| AGPKEDVDEEDFQEYLKEVK | 2 | 2367.11 | 754.44 | 0.00 | 31.11 |
| AVANFSHTLK | 0 | 1086.58 | 0.00 | 1622.07 | 90.83 |
| EMTTDTVQIVYK | 0 | 1426.70 | 0.00 | 979.29 | 155.57 |
| EMTTDTVQIVYKR | 1 | 1582.80 | 0.00 | 1429.46 | 64.12 |
| ESGGYGVIVPQMRD | 1 | 1506.71 | 0.00 | 3136.36 | 151.98 |
| EVDVTLSAR | 0 | 988.52 | 861.86 | 2288.58 | 109.71 |
| IYPANVMR | 0 | 962.50 | 0.00 | 1429.96 | 76.85 |
| KLPVTDAIK | 1 | 983.60 | 0.00 | 1642.20 | 76.83 |
| LYVEEK | 0 | 779.41 | 0.00 | 425.26 | 91.58 |
| MDTQLFDKEEQLQR | 1 | 1779.85 | 1645.05 | 7534.93 | 110.98 |
| QFAELNR | 0 | 876.45 | 0.00 | 689.38 | 94.16 |
| VEVTIYTLR | 0 | 1092.62 | 0.00 | 3034.69 | 81.55 |
|  |  |  | 3261.3 | 30295.7 |  |
